# Cochlear Implantation in Elderly Patients with Residual Hearing

**DOI:** 10.3390/jcm10194305

**Published:** 2021-09-22

**Authors:** Farnaz Matin, Eralp-Niyazi Artukarslan, Angelika Illg, Anke Lesinski-Schiedat, Thomas Lenarz, Marie Charlot Suhling

**Affiliations:** Otorhinolaryngology Department, Head and Neck Surgery, Hanover Medical School, Carl-Neuberg-Str. 1, 30625 Hannover, Germany; Artukarslan.Eralp-Niyazi@mh-hannover.de (E.-N.A.); illg.angelika@mh-hannover.de (A.I.); lesinski-schiedat.anke@mh-hannover.de (A.L.-S.); lenarz.thomas@mh-hannover.de (T.L.); mc_jurawitz@gmx.de (M.C.S.)

**Keywords:** cochlear implant, elderly patients, electric-acoustic-stimulation, hearing preservation

## Abstract

This retrospective study aimed to investigate the range of hearing levels in a cochlear implant (CI) elderly population receiving electric-acoustic-stimulation (EAS) or electric-stimulation (ES) alone. The investigation evaluates the degree of hearing preservation (HP) and the speech comprehension resulting from EAS or ES-only to identify audiometric factors that predict adequate EAS and ES use. We analyzed the pure tone audiometry and speech perception in quiet and noise preoperatively and 12-months after activation of 89 elderly adults (age of 65 years old or older), yielding in total 97 CIs. Thirty-two (33.1%) patients were potential EAS candidates preoperatively, of which 18 patients used EAS at the time of first fitting and the other 14 patients continued to use their residual hearing for EAS at 12-months. Post-treatment, patients with EAS system and ES-only users’ with longer electrodes showed better results in monosyllable word scores in quiet than ES-only users with shorter electrodes. A similar trend was revealed for the speech recognition in noise. Patients with an EAS system benefit from maintaining their natural residual hearing. Nevertheless, strict preoperative patient selection is warranted particularly in elderly patients, in whom the hearing thresholds for EAS indication differ slightly from that in younger adults.

## 1. Introduction

Sensorineural hearing loss (SNHL) is one of the most prevalent chronic diseases among older adults. About one-third of the population in the United States between 65 and 75 years is affected by some degree of SNHL, and the prevalence is estimated to affect more than 50% in the age group 75 years and older [1,2,3]. Similarly, in Germany more than 30% of the population aged between 60 and 69 years is diagnosed with hearing loss. This prevalence increases to approximately 40% and 55% in individuals between 70–79 years and over 80 years, respectively [4,5]. Demographic trends in the minority world [6]—previously known as the ‘developed world’—reveal that the older population grows faster than the total population. As the world’s population progressively grows older, the prevalence of hearing impairment will increase as well [2]. Moreover, previous studies have demonstrated that SNHL is associated with impairments in cognitive function and reduced quality of life [7,8].

For patients with a severe-to-profound SNHL, independent of age, cochlear implants (CI) are the most effective treatment [9]. Improvements in CI technology have led to better performance, with consequent expansion on indication criteria that include wider ranges of SNHL [10]. Hence, patients with partial deafness or profound hearing loss in only the high frequencies—i.e., residual hearing in the low frequencies, benefit from receiving a CI [11]. Specifically, these patients benefit from electric-acoustic-stimulation (EAS) or hybrid systems. EAS consists of a single device that provides a combination of (a) an acoustic amplification in the low frequencies via a HA, which enables use of natural hearing in patients with very good residual hearing, and (b) electric-stimulation (ES) to the high frequencies provided by the CI electrode array [12]. EAS has significant benefits, especially in challenging listening environments [13]. To be able to use an EAS configuration, residual hearing must be preserved after CI surgery.

Due to increased surgical experience and less traumatic electrodes, the chances of hearing preservation (HP) have improved remarkably. Surgical techniques have been developed to increase HP’s chances and reduce cochlear damage intraoperatively [14]. These techniques include gentle puncture and incision of the round window membrane, application of steroids before electrode insertion to prevent inflammation, and slow electrode array insertion via the round window into the scala tympani [1]. Likewise, softer electrode designs with thin diameters, lower stiffness, and different lengths allow the preservation of substantial residual hearing during and after implantation and allow adjustment of the electrode length to the given cochlear length of the individual patient [14,15].

Many studies underlined the influence of the electrode length on HP, showing that the risk of significant postoperative hearing loss (HL) is related to electrode length [12,16]. In particular, the studies showed that longer electrodes are associated with poorer HP. Recently, the concept of partial insertion of longer atraumatic electrodes has been published for HP surgery. This concept allows the possibility of revision surgery in the case of HL progression to insert the electrode deeper [16].

To date, studies have compared the results of different age groups altogether and the impact and postoperative performance of EAS, has not yet been studied placing a special emphasis in the elderly population. Our retrospective study sought to establish a treatment plan for elderly population with a high-frequency severe-to-profound hearing loss but good low-frequency thresholds (i.e., residual hearing). Another aim of this study was to determine their minimum amount of residual hearing required to achieve a speech-perception benefit from EAS.by (a) assessing the degree of hearing preservation with various thin flexible lateral-wall electrodes; (b) analyzing the speech comprehension of ES-only and EAS users 12-months after surgery; and (c) comparing the pre-and postoperative residual hearing and speech comprehension of ES-only and EAS users. The latter would then provide information about the indication criteria for EAS in elderly patients.

## 2. Material and Methods

### 2.1. Subjects

This retrospective study was carried out in an academic tertiary hospital for CI and otosurgery based in Germany. Data of 89 patients with 97 CI, aged 65 years and older at implantation, who received a CI in our institution from May 2009 until July 2017, were collected. All subjects had postlingual uni- or bilateral severe-to-profound SNHL in the high frequencies and some residual hearing in the low frequencies. To determine the amount of residual hearing that would lead to the best benefit with EAS in elderly patients, subjects with a preoperative air-conduction threshold better or equal 80 dB at 250 Hz on the implanted side were investigated in the study.

### 2.2. Array Choice

The patients were implanted with thin, flexible lateral-wall electrode arrays (TFEA) of different lengths depending on the amount of their preoperative low-frequency residual hearing and cochlear length. The audiometric inclusion criteria for implantation and electrode choice have previously been described [10,14].

If patients had significant residual hearing in the low frequencies, meaning 65 dB HL or better at 500 Hz, the patients were informed about the possibility to use EAS by using their preserved residual hearing postoperatively in combination with the CI. If the patients decided on the option of an EAS use, the surgeon decided for a shorter electrode insertion depth intending to preserve the patient’s residual hearing. For an shorter insertion depth either shorter electrode was chosen (i.e., TFEA 16, TFEA 20, or TFEA 24) or a partial insertion of a longer electrode (TFEA 24 and TFEA 28) (MED-EL^®^, Innsbruck, Austria) was performed. Before developing the concept of partial insertion in our clinic, shorter electrodes were selected. Afterwards partial insertion was the preferred treatment option as it allows for deeper insertion of the electrode to provide a higher cochlear coverage for ES only, if hearing is lost after surgery or over time.

Some subjects who were not typical EAS candidates asked for full preservation of their residual hearing. For that reason, these subjects received shorter electrodes on how precious their residual hearing was to them, with the assumption that the shorter array gives better preservation than the longer one.

### 2.3. Surgery

The CI Surgery was performed using the standardized surgical technique at our department [17]. Briefly, a mastoidectomy with opening of the facial recess and exposure of the round window niche was drilled. It was followed by gentle puncture and incision of the round window membrane and insertion of the electrode array into the scala tympani, fixation of the electrode array in a bone slit of the facial recess, and fixation of the internal part of the device. Out of the 97 implantations, 93 electrodes were fully inserted using the round window approach, and four were partially inserted. The fully inserted implanted devices were: TFEA 16 (*n*: 3), TFEA 20 (*n*: 18), TFEA 24 (*n*: 21) and TFEA 28 (*n*: 51). The partially inserted devices were: TFEA 24 (*n*: 2), TFEA 28 (*n*: 2).

### 2.4. Audiometric Assessment

Each patient’s audiometric air conduction thresholds were registered by pure-tone audiometry (measuring the pure-tone average (PTA) low at 125, 250, 500, 1000, and 1500 Hz) preoperatively to define a baseline for each subject. Pure tone audiometer limits were 95 dB at 125 Hz, 100 dB at 250 Hz and 110 dB at 500 Hz to 1500 Hz. Postoperative air conduction thresholds were measured at FF and the 12-months follow-up visit. Preoperative to postoperative change in PTA low for each individual was reported. For analyzing the HP, the postoperative PTA for each visit of the individual subject was plotted against the preoperative PTA. The degree of loss of residual hearing after surgery was divided into three groups: PTA shifts ≤ 15 dB, PTA shift > 15 to ≤30 dB, and PTA shift > 30 dB as described previously [2,14].

For HP analysis, the subjects were postoperatively divided depending on the electrode array and electrode insertion they received: partially inserted electrodes (TFEA 24 and TFEA 28) (*n* = 4); subjects with a TFEA 16 electrode (*n* = 3); subjects with a TFEA 20 electrode (*n* = 19); subjects with a TFEA 24 electrode (*n* = 19) and subjects with a TFEA 28 electrode (*n* = 45).

### 2.5. Fitting

The EAS-System was offered to all patients, who were intended to use EAS based on the preoperative hearing thresholds and their individual counseling and was fitted at the appointment of the first fitting (FF) of the CI, approximately four weeks after implantation.

All patients used an individual earmold with tubing for the acoustic amplification, manufactured by a hearing aid acoustician. The acoustic part of the EAS-System and the crossover-frequency between electric and acoustic stimulation were fitted based on the air-conduction thresholds measured at the FF appointment. Initially, the crossover-frequency between electric and acoustic hearing was set at the audiometric frequency at which the HL exceeds 65 dB HL as recommended by the software. In *n* = 6 cases, the crossover frequency was slightly changed to higher frequencies based on patients’ feedback or to better match with the place frequency of the apical contact. In *n* = 6 patients the postoperative residual hearing was below 65 dB HL in all frequencies, so no crossover frequency was recommended by the software, and the crossover was selected based on the individual slope of the audiogram at a hearing loss between 70 to 80 dB HL.

The subjects using ES-only received a map for ES with a default frequency mapping. The applied frequency range was 100 Hz to 8.5 kHz or since the introduction of the software version MAESTRO 7.0 (MED-EL^®^, Innsbruck, Austria) in 2017 70 Hz to 8.5 kHz.

### 2.6. Speech Comprehension Testing

Preoperatively all patients were measured monaurally in unaided condition using headphones at 60 dB, 80 dB, 100 dB, and if accepted by the patient 110 dB to determine maximum speech understanding at optimal sound pressure level (dBopt) in the ear to be implanted.

The evaluation of postoperative speech recognition was assessed using monosyllabic word test (Freiburg Monosyllabic Word Test (FMWT)) in quiet at 65 dB sound pressure level (SPL) as well as the speech recognition in the German language Hochmair-Desoyer, Schulz, Moser Sentence Test (HSM) in quiet at 65 dB SPL and in noise at 0° azimuth (S0N0) at a 10 dB signal-to-noise ratio (SNR) and post-activation after 12-months of device use. The percentage of correct answers obtained was measured. The tests were conducted with the implanted ear only in the subjects’ everyday listening configuration (EAS or ES). If the patients had a CI or HA on the contralateral side, it was turned off and taken away. Additionally, the contralateral ear was plugged and muffled, in bimodal patients or bilateral EAS patients to eliminate the influence of the residual hearing or masked for a contralateral hearing loss better than 30 dB HL. If not possible, the test was performed using the direct input of the processor, which was the case for *n* = 6 patients

Subjects using EAS were tested in EAS-mode; ES-only users were tested with implanted ear open.

For speech test analysis, the subjects were postoperatively divided into four groups whether they used the EAS system or ES only and depending on the electrode array they used for ES only users. The first group consisted of EAS users (*n* = 11), which were treated with shorter electrodes of different lengths (TFEA 16, TFEA 20, or TFEA 24 electrode) or partially inserted longer electrodes (TFEA 24, TFEA 28); the second group consisted of ES-only users with a TFEA 20 electrode (*n* = 10); the third group consisted of ES-only users with a TFEA 24 electrode (*n* = 17), and the fourth group consisted of ES-only users with a TFEA 28 electrode (*n* = 40).

Intragroup comparison of the preoperative results to 12-months FMWT results was carried out per group.

### 2.7. Comparison of Subjects Using ES-Only to Subjects Using the EAS System

The outcomes of the ES-only users and subjects using the EAS-System under everyday listening condition were compared. The medians, minimum and maximum values at 12-months, and the following patient factors, pre-and postoperative, were determined: age, duration of hearing aid use, and the preoperative tone and speech audiometric values and postoperative audiometric thresholds at 12-months.

### 2.8. Data Analysis

Unfortunately, due to the retrospective nature of the data analysis, we could not evaluate all measurements at all time points for all patients. Subsequently, the number of subjects varied between tests. As a consequence, each figure contains the corresponding number of patients for each condition.

To evaluate the speech comprehension data, the four groups were treated as independent samples. The groups were tested for variance homogeneity using the Levene-Test. In case of variance homogeneity ANOVA was used for group comparisons. If variance homogeneity was not fulfilled the Brown Forsythe test was used for group comparisons. The Bonferroni corrected post-hoc test was applied for pairwise comparison.

To evaluate the difference of patient’s factors, pre-and postoperative and outcomes between ES- and EAS users at 12-months, the Mann-Whitney U Test for independent samples was used, because the data were not normally distributed.

The Wilcoxon signed-rank test for dependent samples was used to compare speech data from the preoperative to the postoperative condition for each group.

Statistical significance was set to *p* < 0.05 (* *p* < 0.05, ** *p* < 0.01, *** *p* < 0.001). All data were analyzed statistically using SPSS (IBM, SPSS Statistics 22, Chicago, IL, USA).

The responsible ethics committee approved the protocol for using the patient’s data for this retrospective study (Project identification code 7657-2018). Due to the retrospective design, no written information was given to the patients of the study group. All patient data were anonymized and de-identified prior to the retrospective analysis.

## 3. Results

### 3.1. Subjects

A total of 89 patients, 47 female and 42 male, aged 65 years and older, were implanted at our center. The mean age at implantation was 73 years (65–89-year-old). The patient’s demographic data (sex, side of implantation, type of electrode array, etiology of SNHL, and use of HA in the implanted ear) are summarized in Table 1.

### 3.2. Electric Acoustic Stimulation Users

Thirty-two patients fulfilled the audiometric EAS indication preoperatively. Eight-teen elderly patients tried the EAS-System, 14 subjects were EAS users until 12-months postoperative

Two patients of those 4 patients, who were no EAS users anymore at 12 months, had suffered from a pre-to postoperative hearing loss > 30 dB and had only minimal residual hearing already at time of first fitting. The EAS system was fitted to try if the acoustic amplification would give at least some additional subjective benefit, which was not the case and they stopped using EAS. One patient with a TFEA 24 electrode used the EAS program additionally to ES, but preferred ES-only. One patient received and EAS-system and used it regularly but was lost to follow-up after the 3 months appointment.

### 3.3. Hearing Preservation

**Figure 1:**
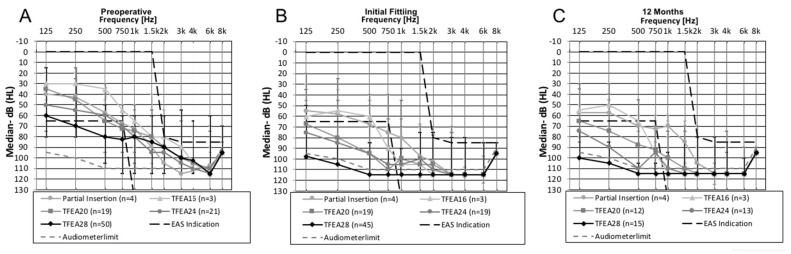
Pre- and postoperative median hearing levels, minimum and maximum for all 97 ears, divided into groups: (**A**) preoperative, (**B**) FF, (**C**) 12-months after FF.

The patient group with partially inserted electrodes showed median HL averaged across the frequencies 125–1500 Hz of 6.5 dB after 12-months, which was the smallest median value compared to the other groups with fully inserted electrodes of different lengths. An increase of median HL were observed for TFEA 16 (*n* = 3) from 19 dB HL at FF, to 23 dB HL at 12-months (*n* = 3), for TFEA 24, from 21.3 dB HL at FF (*n* = 19), to 32.5 dB HL at 12-months (*n* = 13) and for TFEA 28, from 31.7 dB HL median HL across all frequencies at FF (*n* = 45), to 34 dB HL at 12-months (*n* = 15). Hearing was preserved over time for the subjects implanted with a TFEA 20 with a decrease in median HL from 21.3 dB HL at FF (*n* = 19) to 17.3 dB at 12-months (*n* = 12) (Table 2 and Figure 1).

### 3.4. Speech Comprehension

#### 12 Month Interval

1. FMWT Scores: The median for EAS users was: 45.00%. For the ES-only groups, the median was: TFEA 20 ES: 32.50%, TFEA 24 ES: 50.00%, and TFEA 28 ES: 55.00%.

**Figure 2:**
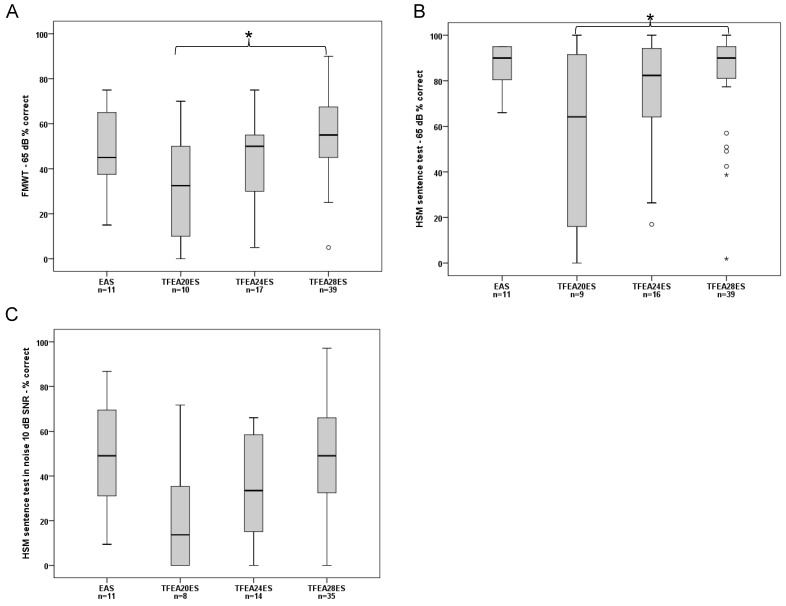
Median, interquartile, and minimum and maximum scores after 12-months: Statistical significances are marked with * for *p* < 0.05 **A**) FMWT, (**B**) HSM sentence in quiet, (**C**) HSM sentence in noise.

There was a significant effect of group on FMWT Scores (ANOVA, F (3, 73) = 3.741, *p* = 0.015. Post-hoc tests showed a significant difference only between TFEA 20 ES users and TFEA 28 ES users (Bonferroni corrected post-hoc test: *p* = 0.014). The pairwise comparison of the other groups did not show significant difference (Figure 2A).

2. HSM in quiet: The median for EAS users was: 90.00%. For the ES-only groups, the median was: TFEA 20 ES: 64.15%, TFEA24 ES: 82.36%, and TFEA 28 ES: 90.00%. A significant effect of group was detected for the HSM in quiet (Brown-Forsythe, F (3, 17.803) = 3.634, *p* = 0.033). Bonferroni corrected post-hoc tests revealed a significant difference only between TFEA 20 ES users and TFEA 28 ES users (*p* = 0.004). The pairwise comparison of the other groups did not show significant difference (Figure 2B).
3. HSM in noise: The median for EAS users was: 49.05%. For the ES-only groups, the median was: TFEA 20 ES: 13.68%, TFEA 24 ES: 33.49%, and TFEA 28 ES: 49.05%. There was a significant effect of group on the HSM in noise (ANOVA, F (3, 54) = 2.785, *p* = 0.048. However, Bonferroni corrected post-hoc tests did not show significant difference for pairwise comparisons (Figure 2C).

### 3.5. Comparison of the Speech Comprehension: Preoperative with Headphones versus Postoperative EAS/ES-System

#### 12-Months Interval

**Figure 3:**
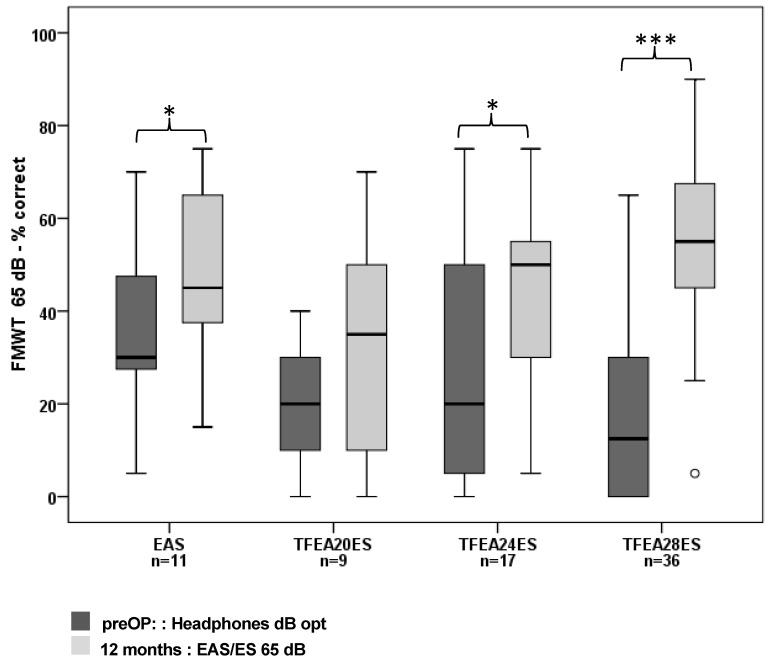
Median, interquartile, minimum and maximum FMWT scores preoperatively (unaided at optimum dB SPL, tested with headphones) and 12-months postoperatively (i.e., CI use). Significant differences are marked with * for *p* < 0.05, and *** for *p* < 0.001.

The within group comparison of the preoperative FMWT unaided at optimal dB SPL when tested with headphones and 12-months postoperative FMWT results after CI use showed significant differences between the pre- and postoperative condition for all groups EAS group (Wilcoxon signed-rank test: T = 10.5, *p* = 0.045,), TFEA 24 ES group (Wilcoxon signed-rank test: T = 21, *p* = 0.015) and TFEA 28 ES group (Wilcoxon signed-rank test: T = 20.5, *p* < 0.001) (Figure 3).

### 3.6. Comparison of Subjects Using ES-Only to Subjects Using the EAS System

Table 3 summarizes age, hearing aid use, pre-and postoperative audiometric outcomes for all subjects using ES-only with subjects using the EAS-System at 12-months post activation.

No difference was identified for age and duration of hearing aid use between the groups. For the preoperative and postoperative hearing thresholds between ES and EAS, significant differences were found along with all analyzed frequencies (125 Hz, 250 Hz, 500 Hz, 750 Hz) and the PTA (*p* < 0.001).

In the EAS group, the median preoperative hearing thresholds at 500 Hz, was 52.5 dB HL with a range of 30 dB HL to 80 dB HL, meaning that some patients did not fulfill 65 dB HL criteria preoperatively. For 125 Hz and 250 Hz, all patients in the EAS group fulfilled the preoperative EAS indication, and the median values were with 45 dB HL for 125 Hz and 42.5 dB HL for 250 Hz 20 dB HL below the indication.

The median postoperative values for 125 Hz and 250 Hz were 50 dB HL and 57.5 dB HL also below 65 dB HL.

In the ES group, the median preoperative hearing thresholds were significantly worse than the preoperative EAS hearing thresholds. The postoperative values for 125 Hz, 250 Hz, and 500 Hz were median 90 dB HL, 105 dB HL, and 115 dB HL, also significantly worse than the postoperative hearing thresholds than in the EAS group.

The preoperative maximal speech perception with headphones at dBopt, which was in median 100 dB for both groups, was significantly different between the ES (Median: 17.5%) and EAS groups (Median: 32.5%) (Mann-Whitney, U = 216.5, *p* = 0.020). All patients using EAS at 12 months had a least some speech perception with headphones preoperatively.

## 4. Discussion

In the minority world [6], CI has become a safe and effective intervention for patients with uni- or bilateral severe-to-profound SNHL when HAs do not provide sufficient benefit [18]. Hence, adult CI users have an already long audiological history by the time they receive CI counselling and decide to undergo CI surgery. Typically, they have worn HAs or used other hearing technologies for a long time until implantation [19]. Importantly, when patients present with a significant amount of natural low-frequency hearing, there is reportable fear of losing their residual hearing after implantation. They question whether their hearing ability and overall comprehension experience after CI will improve compared to their current hearing status.

The present study sought to evaluate audiological outcomes in elderly patients with residual hearing suitable for an EAS system in order to provide this specific patient population with better counselling that includes well-grounded predictions or expectations, and ultimately a better treatment. Our retrospective study is unique in that it determines for the first time in a large cohort of 97 elderly (>65 years of age) CI recipients the degree of postoperative HP rates with outcome comparison of these EAS and ES-only users. The main strengths of this study lie in the large number of cases included, the use of the same atraumatic arrays of different lengths, and the homogeneity of the series of patients: operated by the same team of experienced CI surgeons and underwent the same clinical assessment up to 12-months post activation. We chose this follow-up interval due to the finding of Lenarz et al. [20] that one year after implantation most postlingually deafened adults, independent of age, reach a stable plateau phase in their CI performance.

### 4.1. HP in Chronological Sequence

Regarding HP at the FF, the median HL for the partial insertion was 10 dB, for the TFEA 16 19 dB, for the TFEA 20 21.25 dB, for the TFEA 24 21.25 dB, and for the TFEA 28 31.7 dB. In the group of patients implanted with TFEA 16, TFEA 24, and TFEA 28, the median HL increased to 12-months post activation. In the group of patients implanted using partial insertion and TFEA 20, the median HL decreased 12-months post activation. However, a direct comparison of the median HL over time is difficult across all electrodes because the number of subjects undergoing the audiometric assessment at the follow-up dates differed greatly (Table 2).

Moreover, the presented cohort is rather uniform in the criteria for HP, type of electrode arrays, and implemented standardized follow-up protocols; this uniformity further facilitated reliable data comparison with other CI recipients falling in different age groups, as reported in previous studies from our group. Suhling et al. [14] measured in a group of 120 subjects with a mean age of 57.33 years old (18–90 years old) different and (slightly) better results for HP at the time of FF: the median HL for the TFEA 20 was 17.5 dB, for the TFEA 24 20 dB and for the TFEA 28 24 dB. In addition, the same study measured at 12-months postoperative a stable median HL at 15 dB for TFEA 20 recipients and 19.4 dB for TFEA 24 recipients, but an increasing median HL to 32.5 dB for TFEA 28 recipients. These HP findings over time are similar to the results of the TFEA 20 and TFEA 28 recipients in our study for the interval 12-months postoperative (Table 2).

Our study demonstrates that HP in most elderly CI recipients is feasible 12-months post-activation with shorter electrode arrays—despite concerns about cochlear fragility in these patients. The proportion of the subjects with good HP (≤15 dB) was greater with the shorter insertion (75% partial insertion; 33.3% TFEA 16; 41.7% TFEA20) (Table 2). The loss of residual hearing was slightly greater in elderly patients than in other studies of our group it but this difference was no longer observed at the 12-months postoperative mark [14]. Age-related degenerative changes occurring in the auditory pathways might be the reason for greater HL in the lower frequencies [21,22]. Cadaveric studies of Nadol et al. [23] have demonstrated age-related effects on the peripheral auditory system, specifically, decreased spiral ganglion cell counts within the cochlea. Also, on a cellular level, the aging brain is associated with decreased synaptic density and dendritic cell numbers, which may have implications for neural plasticity [24].

### 4.2. Speech Comprehension

ES-only subjects implanted with the shorter arrays, i.e., TFEA 20 and TFEA 24, had higher degrees of residual hearing before surgery compared to the TFEA 28 recipients. We observed that 12 months after CI activation, the HSM in noise test scores of subjects with the TFEA 20 ES-only or TFEA 24 ES-only users who lost usable residual hearing were barely half of those of the TFEA 28 ES group (Figure 2). Interestingly, a previous study also from our group [13] showed slightly better speech comprehension results than this study. However, their study included follow-up data of only up to six-months, challenging in this sense a direct comparison with our longer follow-up findings. In their study, Büchner & Illg et al. described up to 10% better results in all speech comprehension tests, especially for EAS and TFEA 20 ES-only users. The results of the TFEA 24 ES-only and TFEA 28 ES-only users were comparable. However, in both our and Büchner’s & Illg’s evaluation, the TFEA 28 ES-only users performed significantly better in the speech test than subjects with shorter electrodes. The EAS users achieved significantly better results, especially in terms of speech comprehension tests in noise. Anecdotally, we have seen a strong age dependency in EAS performance in speech comprehension tests in noise [25]. Patients below the age of 70 show up to 20% better results in speech comprehension tests in noise than EAS users over 70 years of age [25].

Our observation of worse HP in elderly patients corroborates the recently published study by Bourn et al. [26]. They analyzed the HP six-months post-activation of recipients 72 years and older using the HP classification by Skarzynski et al. [27]. HL in the low frequencies was greater when longer electrodes (i.e., TFEA 24 and TFEA 28) were inserted. In fact, speech comprehension scores were especially lower in patients who received TFEA 24 electrodes. Furthermore, TFEA 28 ES-only users achieved better speech comprehension scores than TFEA 24 ES-only users (Figure 2), despite their worse HP. This finding is in agreement with the work of Buchman et al. [28] who examined speech understanding in subjects with seven MED-EL STANDARD (length 31.5 mm) or six MEDIUM (length 24 mm) electrodes, showed that insertion with longer electrodes resulted in better speech perception performance, at least in the early post-activation period. Our results from the ES-only users with no usable acoustic hearing show that longer arrays significantly increase speech understanding (Figure 2).

An explanation for better speech comprehension in the subjects with deeper insertion angles is attributed to a wider cochlear coverage leading to electrically stimulation of the low frequency—apical region in the cochlea [12,13,14]. Also, the larger spacing of electrode contacts seen in the longer electrodes might lead to less crosstalk or channel interaction between adjacent contacts. Subsequently, less channel interaction improves spectral resolution, which results in better speech understanding in noise [13,29].

As the outcomes clearly show, postoperative speech comprehension improved significantly in all elderly CI recipients compared to their preoperative speech perception using HAs. There are additional advantages for EAS users regarding music perception, separating individual talkers from each other, and listening to the intended target speaker [30,31,32]. This advantage seems to derive from their natural acoustic hearing or residual hearing in the low frequencies [33,34]. By improving verbal communication, CI restores the possibility of social networking in this age group and positively affects social activity that contributes to better cognitive function [35]. Moreover, Knopke et al. [36] reported benefits in quality of life and improvement of stress related to tinnitus in elderly CI recipients.

As reflected in the FMWT scores postoperatively (Figure 3) EAS might not give a huge benefit over conventional ES-only with longer electrodes. Our findings suggest elderly patients should be carefully selected for EAS use, paying particular attention to the preoperative frequencies 250 Hz and 500 Hz. Compared to the hearing thresholds for EAS indication of younger adults, the hearing levels for sufficient long term EAS use of elderly patients differ up to 20 dB in the frequencies 125 Hz, 250 Hz, and 500 Hz that need to be accounted for. Elderly patients with preoperative hearing worse than 42.5 dB at 250 Hz and below 52.5 dB at 500 Hz do not have sufficient benefit from EAS (Table 3) and should rather receive longer electrodes for ES-only use. The preoperative unaided FMWT results are also important, and the results should not be below 32.5% (Table 3). Based on these findings the insertion depth has to be considered carefully on a case-by-case basis, especially in elderly patients with slightly different criteria for EAS selection. Moreover, the concept of partial insertion of longer electrodes can be used with the option for deeper electrode insertion if the hearing loss of the residual hearing progresses or where there is a disadvantage of EAS [16].

The above-mentioned factors can be considered preoperatively to select the best treatment solution for elderly patients, as we have provided better understanding on the expected postoperative development of residual hearing and hearing performance, especially with EAS.

## 5. Conclusions

The preservation of low frequency residual hearing is feasible in elderly CI recipients. The EAS-System for elderly patients is beneficial for natural acoustic hearing, but it needs strict patient selection preoperatively. The preoperative hearing thresholds for sufficient long term EAS use differ up to 20 dB in the frequencies 125 Hz, 250 Hz, and 500 Hz from the younger adults that need to be accounted for. If the residual hearing in the low frequencies is at the limit of the EAS criteria, then ES-only stimulation with longer electrodes leading to deeper insertion enhances the postoperative speech understanding should be preferred in this special patient population.

## Figures and Tables

**Table 1:** Patients’ demographics of 89 patients and 97 CI electrode arrays.

Age at Implantation (Mean ± SD)	73	± 6
**Sex**	%	*n*
female	52.8%	47
male	47.2%	42
**Laterality**		
right ear	54.6%	53
left ear	45.4%	44
bilateral	9.0%	8
unilateral	91.0%	81
**Electrode array**		
TFEA 28	52.6%	51
TFEA 28 partial insertion	2.1%	2
TFEA 24	21.6%	21
TFEA 24 partial insertion	2.1%	2
TFEA 20	18.6%	18
TFEA 16	3.1%	3
**Etiology of SNHL**		
Idiopathic SNHL	71.1%	69
Acute SNHL	18.6%	18
Ménière’s disease	4.1%	4
Acoustic neurinoma	2.1%	2
Otosclerosis	2.1%	2
Acoustic trauma	2.1%	2
**Use of HA in the implanted ear**		
until the surgery	72.2%	70
until 12-months preoperatively	4.1%	4
more than 12-months preoperatively	13.4%	13
no use of HA	10.3%	10

**Table 2:** Postoperative hearing loss (HL) with different partial inserted electrode arrays and full inserted electrode arrays at the time of the first fitting (FF) and 12-months (12 M).

-	-	≤15 dB	>15 HL ≤ 30 dB	>30 dB
Partial Insertion				
TFEA 24/TFEA 28				
FF (*n* = 4) (average insertion depth: 17.5 mm)	10.0	3 (75.0%)	1 (25.0%)	0 (0%)
12 M (*n* = 4) (average insertion depth: 17.5 mm)	6.5	3 (75.0%)	1 (25.0%)	0 (0%)
TFEA16				
FF (*n* = 3)	19.0	1 (33.3%)	1 (33.3%)	1 (33.3%)
12 M (*n* = 3)	23.0	1 (33.3%)	1 (33.3%)	1 (33.3%)
TFEA20				
FF (*n* = 19)	21.3	7 (36.8%)	4 (21.1%)	8 (42.1%)
12 M (*n* = 12)	17.3	5 (41.7%)	3 (25.0%)	4 (33.3%)
TFEA24				
FF (*n* = 19)	21.3	5 (26.3%)	8 (42.1%)	6 (31.6%)
12 M (*n* = 13)	32.5	3 (23.1%)	3 (23.1%)	7 (53.8%)
TFEA28				
FF (*n* = 45)	31.7	3 (6.7%)	16 (35.6%)	26 (57.8%)
12 M (*n* = 15)	34.0	0 (0%)	5 (33.3%)	10 (66.7%)

**Table 3:** Comparison of ES-only users and EAS users at 12-months regarding age at implantation, hearing aid duration, and preoperative FMWT results unaided at optimum dB SPL when tested with headphones and preoperative and 12-months post-activation at hearing levels for 125 Hz, 250 Hz, and low-frequency PTA median, minimum and maximum.

Variable	ES-Only User 12 M-Median (Min; Max)	EAS User 12 M-Median (Min; Max)	Mann Whitney U-Test (U-Value, *p*-Value)
age in years	72.5 (65; 89) *n* = 74	71.0 (66; 83) *n* = 14	U = 441.0, *p* = 0.379
hearing aid duration in years	10.0 (1; 50) *n* = 56	11.0 (7; 30) *n* = 7	U = 224.0, *p* = 0.555
preop FMWT dB opt Headphones in %	17.5 % (0; 75) *n* = 70	32.5 % (5; 70) *n* = 14	U = 683.5, *p* = 0.020
preOP AC 125 Hz	55.0 dB HL (15; 80) *n* = 58	45.0 dB HL (15; 55) *n* = 11	U = 186.0, *p* = 0.028
preOP AC 250 Hz	65.0 dB HL (20; 80) *n* = 74	42.5 dB HL (15; 65) *n* = 14	U = 221.5, *p* = 0.001
preOP AC 500 Hz	75.0 dB HL (35; 110) *n* = 74	52.5 dB HL (30; 80) *n* = 14	U = 177.5, *p* = 0.000
preOP PTA LF	74.0 dB HL (46; 93) *n* = 74	60.5 dB HL (44; 75) *n* = 14	U = 199.5, *p* = 0.000
postop FA AC 125 Hz	82.5 dB HL (40; 100) *n* = 62	55.0 dB HL (20; 75) *n* = 13	U = 77.5, *p* = 0.000
postop FA AC 250 Hz	100.0 dB HL (40; 105) *n* = 66	57.5 dB HL (25; 85) *n* = 14	U = 47.0, *p* = 0.000
postop FA AC 500 Hz	110.0 dB (40; 115) *n* = 67	70.0 dB (45; 100) *n* = 14	U = 36.5, *p* = 0.000
postop FA PTA LF	101.0 dB (47; 113) *n* = 67	69.5 dB (59; 98) *n* = 14	U = 73.5, *p* = 0.000

Abbreviations: AC = Air conduction; PTA = pure-tone average; LF = low frequencies; 12 M = 12-months.

## Data Availability

The data is presented within the article.
